# On the Proportion of the Subjects Bitten by Mad Animals, Who Become Affected with Hydrophobia

**Published:** 1852-12

**Authors:** 


*On the Proportion of the Subjects bitten by Mad Animals, who become affected with Hydrophobia.* By Professor Renault.—M. Renault, Clinical Professor at the Veterinary School at Alfort, has recently presented to the Academy a valuable Report, adverse to the claim again recently set up for the prophylactic power of mercurial inunction in hydrophobia. As this claim has been over and over again refuted, we should not have adverted to the subject, but for the valuable incidental matter introduced by the Professor into his Report. He observes, that before we can receive the numerous cases which have been published of the preservative power of mercurial salivation, it is necessary,—1. That it should be placed beyond all doubt that the animal inflicting the bite was really mad; and that all the persons supposed to have been preserved have really been exposed to the infection. On examining the histories of these cases, however, such proof is quite defective, as in some of those related—e.g., by Ehrmann and Audry, the persons had not been bitten at all, but had merely lain or drank with those who had, or had wiped away their saliva.—2. Next, when persons have been really bitten, and the animal has been really mad, we must be satisfied that the poison has been deposited in the wound. But numbers of the persons referred to have been bitten through their clothes, and every one knows how small a proportion of such acquire hydrophobia, even when no treatment at all has been employed.—3. But even when the poison has been duly deposited in the wound, to admit the preservative power of the mercury, we must allow that all such persons would necessarily suffer from hydrophobia if left to themselves. Numbers of cases contradictory of this are, however, on record. But although all careful observers admit this, the *proportion* of such as escape has never been duly investigated; and the subject being one of the highest interest, M. Renault takes the opportunity of communicating the results of the investigations he has been conducting upon it at Alfort since 1828.

To prevent confusion, he arranges the cases he has collected in two categories. (A.) Cases of dogs or other animals accidentally bitten by others, either mad or supposed to be so, and sent by the police to Alfort, to remain under inspection. Of 224 dogs so brought, between 1827 and 1837, which continued under observation for more than two months, without undergoing treatment, 74 or nearly one-third became mad, and 130 or two-thirds exhibited no symptoms. It is evident, however, that these cases do not afford any measure of the activity of the virus; for the dog that caused the bite may not always have been mad, the bites could not always be verified, and the hair of the animal may have prevented the poison from penetrating.

(B.) For this reason, another series of facts is adduced. From 1830 to the present time, certain dogs known as really mad have been made to bite, at the Clinical School, other dogs or herbivora in portions of the surface of delicate structure and devoid of hair, or inoculation has been performed with some of their saliva, collected during the height of the paroxysm. Of 99 dogs, horses, and sheep, so treated, 67 became mad, and 32 continued under observation more than 100 days without any symptom exhibiting itself. Thus, in this category, in which every circumstance favorable to infection was secured no less than one-third of the animals escaped, without undergoing any treatment whatever.

Examining the experience of other clinical professors, M. Renault says, that Professor Rey, of Lyons, has found that among animals who were accidentally bitten in the streets, and then placed under *surveillance*, 1 to 5 of the dogs, and 1 to 4 of the horses, became mad. Of those bitten or inoculated experimentally, 2 to 3 became mad. Of 16 animals accidentally bitten at Toulouse, 5 became mad. Professor Hertwig, of Berlin, states, that of 137 dogs accidentally bitten, 16 only became mad, 121 remained uninjured; while of 25 experimentally infected 10 became mad, and 15 did not. Thus, in both the categories, the proportion affected was sensibly less in Berlin than in France, whether this be owing to climate or other cause.

Thus, taking things at the worst, it results from these observations, made at different epochs, that two-thirds of the animals accidentally bitten, and one-third of those artificially infected, escape.

Still, it must not be supposed that these *mean results* of a great number of observations can be applied when speaking of the bite of a particular dog; for M. Renault has repeatedly observed, that while one dog, evidently mad, bites several others, and only one-sixth or one-seventh of these shall suffer, the virus conveyed from another dog, to all appearance in just the same condition, will infect nearly every animal (five-sixths or six-sevenths.)

Moreover, it is generally believed that the bites of *mad wolves* are oftener followed by hydrophobia than are those of dogs. Of 254 examples of such bites, the histories of which M. Renault has been able to collect in authors, in 164, or nearly two-thirds, hydrophobia followed—the proportion for accidental bites by mad dogs being only one-third. Whether this depends upon the fact, that in the wolf the rabies is oftener spontaneous, or upon the more remarkable one, that this animal almost always bites its victim in the face, neck, or head, is uncertain.—*Bull. de l'Acad.*

[Hydrophobia has recently been very prevalent in France, so that something like a panic has been produced and the journals teem with accounts of supposed remedies. The police have made a terrible onslaught on all wandering dogs; and the exemption of London from the disease, since the prohibition of the employment of dogs as beasts of burden, has been adverted to. It would be desirable to ascertain how far this exemption has extended beyond the metropolis, where alone the dog-act is in operation. How much remains to be cleared up in the history of *rabies canina*, is seen by a communication from Clot-Bey(*L'Union Med.* 1852, No. 94,) in which he states, that while dogs exist in such vast numbers in various parts of Asia and Africa, hydrophobia is of the rarest occurrence. In Egypt where he lived during twenty-five years, he never heard of a case, although the dogs wander abont the towns and villages in vast numbers, under a burning sun panting for breath, ill-fed, and often deprived of water. He thinks that much may be due to the promiscuous intercourse which their entire liberty allows to these animals. In Greece, on the other ha[illegible]d, the disease is excessively prevalent.—*British and [illegible]reign Medico-Chirurgical Rev.*

